# How Is Chronic Pain Managed in Rural Australia? A Qualitative Study Exploring Rural Healthcare Professional and Consumer Experiences

**DOI:** 10.1111/ajr.70000

**Published:** 2025-02-10

**Authors:** Ashley R. Grant, Gill Westhorp, Amelia Mardon, Monique White, Peter D. Hibbert, Emma L. Karran, Christopher Roeger, G. Lorimer Moseley

**Affiliations:** ^1^ IIMPACT in Health University of South Australia: Allied Health and Human Performance Adelaide South Australia Australia; ^2^ Faculty of Arts and Society, RREALI Charles Darwin University: Northern Institute Casuarina Northern Territory Australia; ^3^ NICM Health Research Institute Western Sydney University Westmead New South Wales Australia; ^4^ Independent Consumer Researcher Murray Bridge South Australia Australia; ^5^ Australian Institute of Health Innovation Macquarie University Sydney New South Wales Australia; ^6^ Tumby Bay Medical Tumby Bay South Australia Australia

## Abstract

**Introduction:**

Guideline‐based care for chronic pain is variably provided. Existing data on chronic pain management in Australia come primarily from metropolitan samples. As the initial investigations for a wider needs assessment, we sought to understand how chronic pain is managed in rural Australia, focused on investigating the gap between guideline‐recommended care and provided care.

**Methods:**

We conducted semistructured interviews with rural healthcare professionals who treat patients with chronic pain and rural consumers affected by chronic pain. We asked healthcare professionals what treatments they deliver to patients with chronic pain. We asked consumers to describe the healthcare service providers they had accessed for pain care and the treatments they received from these providers. We utilised content analysis to gain an understanding of what care for chronic pain is being provided and compared these findings to guideline recommendations.

**Results:**

We interviewed 15 healthcare professionals and 27 consumers. Both healthcare professionas and consumers reported minimal use of most first‐line management strategies. We also found differences between healthcare professional and consumer reports of pain care. Healthcare professionals frequently described delivering guideline‐aligned pain care and consumers frequently described receiving care that contradicted guidelines. We identified challenges with rural access to pain care services, including minimal usage of telehealth services.

**Conclusions:**

Given the identified gaps in care, future research may consider ways of improving rural access to pain care services, including investigating ways to increase uptake of telehealth services, and how to shift consumer expectations of pain care.

**Summary**

- What is already known on this subject
  ○ Guideline‐based pain care is variably implemented around the world and in Australia.
  ○ Healthcare professionals practicing in rural, regional and remote Australian settings face additional challenges when providing pain care, including limited resource access.
- What this paper adds
  ○ A report on how pain is managed in rural Australia from the perspectives of a sample of rural healthcare professionals and consumers, including identification of minimal use of first‐line, nonpharmacological management strategies, minimal healthcare professional initiation of referrals to pain care services, and rural consumers accessing pain care with questionable efficacy.

## 1 Introduction

According to the Global Burden of Disease study, four of the top 10 causes for years lived with disability are chronic pain conditions [1]. Chronic pain is defined by the International Association for the Study of Pain (IASP) as ‘pain that persists or recurs for longer than three months’ [2]. Australian guidelines for the management of chronic pain recommend applying a biopsychosocial model, taking a thorough assessment and supporting nonpharmacological self‐management [3]. In practice, it is common across healthcare disciplines for the delivery of pain care to diverge from guideline recommendations [4, 5, 6]. Exposure to nonguideline‐based pain care is associated with increased healthcare costs and nonguideline‐based imaging is associated with increased healthcare utilisation, work absenteeism, surgery and prescription opioid use [7, 8].

Australian studies suggest guideline‐based care for chronic pain is inconsistently provided. The CareTrack Australia study utilised data from 10 492 healthcare encounters by 412 consumers with chronic pain conditions and reported more than a quarter of encounters for these conditions included care that was not consistent with relevant guidelines [9]. Studies reporting on healthcare professional (HCP) management of chronic pain conditions commonly described high rates of imaging and medication prescription, and underutilisation of recommended first‐line nonpharmacological strategies, including supporting increased physical activity and provision of education [10, 11, 12]. Similarly, studies from the consumer perspective have reported underutilisation of guideline‐based nondrug and nonsurgical management options, with more than half of consumers in one study reporting never having tried resistance training, cardio‐respiratory fitness classes or hydrotherapy [13].

Rural settings present unique challenges for the delivery of guideline‐based pain care. Examples of such challenges include limited access to HCPs [14], General Practitioner (GP) shortages [15] and longer wait times than metropolitan areas [16]. The COVID‐19 pandemic increased rural health workforce challenges in Australia due to decreased migration of international medical graduates, who previously filled workforce positions through mandatory placements in rural settings [15]. Limited access to primary, specialist and allied health services in rural settings has been suggested to explain much higher rates of emergency department presentations for low back pain in rural than metropolitan settings [17].

To help inform future programmes targeting the improvement of rural Australian pain care, we conducted a two‐part needs assessment. We sought to investigate the gap between guideline‐recommended care and provided care for chronic pain in rural Australia (Part 1), and to explore factors contributing to the identified gaps and generate recommendations for improving pain care in rural settings (Part 2). In this manuscript, we report on Part 1 of this needs assessment.

## 2 Methods

### 2.1 Research Questions

We sought to answer the following questions: (1) What HCPs are rural consumers accessing for pain care? (2) How are rural consumers accessing pain care services? (3) What referral pathways are rural consumers using for pain care? and (4) How is pain managed by HCPs in rural Australia? We then sought to compare these consumer and HCP reports of how pain is managed in rural Australia to guideline recommendations for pain care.

### 2.2 Research Design

Needs assessments investigate what gaps exist between what is presently happening and what is desired to be happening, explore why these identified gaps exist and then generate recommendations for addressing these gaps [18, 19]. The present manuscript reports on our qualitative investigation into the gap between guideline‐recommended care and provided care for chronic pain in rural Australian settings from the perspectives of an unmatched sample of rural HCPs and consumers. Conducting a qualitative investigation enabled us to gain a deeper understanding of rural Australian pain care from the perspectives of individuals with lived experiences providing and receiving pain care in these settings. A detailed study protocol is available on Open Science Framework (OSF) [DOI 10.17605/OSF.IO/JEPTB]. We generated this protocol prior to starting recruitment, but we waited to register the protocol until we clarified the project plan for Part 2. At the time of protocol registration [February 2023], we had completed interviews with all HCPs and 12 of the consumers.

### 2.3 Ethics

This project was approved by University of South Australia Health Research Ethics Committee (protocol # 204795).

### 2.4 Participants

We recruited rural HCPs through word of mouth and networks of the research team. To be eligible, HCPs needed to have (1) worked in healthcare as a medical or allied health professional in the last 12 months, (2) worked in a rural, regional or remote setting in the last 12 months for a minimum of 3 months and (3) possess experience supporting chronic pain patients in a rural, regional or remote setting in the last 12 months. We separately recruited rural chronic pain consumers through an article in a rural newspaper, promotion via a radio interview and through outreach via Australian organisations that support consumers with pain (i.e., Chronic Pain Australia, Pain Revolution and rural support groups). Rural consumers were eligible if they: (1) lived in a rural, regional or remote setting, (2) had experienced chronic pain, as defined as pain experienced on most days for a period of 3 months or more in the last 12 months and (3) were at least 18 years old.

We classified ‘rural, regional or remote’ settings according to the Modified Monash Model (MM) [20]. This model was chosen because it is the Australian government's system for classifying a location's rurality and it is used to inform health workforce distribution. The rating system ranges from city, MM1, through to very remote communities, MM7 (see File S1 for more details). To be eligible, HCP needed to work in, and consumers needed to reside in, settings classified as MM2 to MM7.

### 2.5 Procedure

We emailed potential participants study information and offered the option of a telephone or an online videoconferencing interview with author ARG. Prior to the interviews, we sent participants an online Qualtrics [www.qualtrics.com/] survey to confirm their eligibility, provide consent and record demographic details. We collected demographic data from HCPs regarding their background and experience to gain an understanding of their clinical expertise. We collected demographic data from consumers that included sex, gender, age, education level, financial and employment status because these factors have been shown to contribute to care seeking and health outcomes for people with chronic pain [21]. We audio recorded and verbatim transcribed all interviews.

### 2.6 Interview Topics

The semistructured interviews followed a topic guide that was generated by the authorship team and pilot tested with two HCPs and one consumer before starting data collection. We sent this topic guide to participants prior to their interview (see File S2). Briefly, we asked HCPs to describe their most utilised management practices for patients with chronic pain, including exploration of their referral pathways to other services. We asked consumers what healthcare services they had accessed for pain care, how they accessed each service (i.e., locally, via telehealth or by travelling), what referral pathway connected them to each service and what treatments they received from each service.

### 2.7 Data Extraction and Analysis

We imported interview transcripts into NVivo 14 [https://lumivero.com/products/nvivo/] for data extraction and analysis. We utilised a qualitative content analysis approach because it is a systematic, rule oriented approach that enabled us to generate meaning by quantifying how often each service provider, referral pathway and management approach was mentioned by HCPs and consumers [22]. This involved structuring deductive category assignment using nominal category systems, with inductive category formation and frequency analysis [22]. At the start of data extraction, we generated a reference document defining categories, with anchor examples and rules for extracting data to each category [22]. We pregenerated categories based on our overarching research questions that each of our interview questions contributed to and generated additional categories when interview data presented additional answers to our research questions (e.g., new HCP or treatment types). For our frequency analysis, we counted the number of times each category was mentioned and the number of different participants who mentioned each category [22]. We extracted quotes to illustrate HCP and consumer responses to our research questions.

During analysis, we organised the HCPs and services consumers reported accessing into categories including: GPs, allied health professionals, specialists, Complementary and Alternate Medicine providers, multidisciplinary pain services and emergency departments. We utilised the Allied Health Professions Australia (AHPA) list of allied health professions [23]. The Complementary and Alternate Medicine providers category encompassed additional providers mentioned in the interviews that did not fit the other categories and where a medicine or therapy was used alongside, or instead of, conventional care.

We tracked both the number of consumers who reported accessing each provider type, as well as the number of different providers of that type the consumer had accessed (e.g., one consumer could report accessing multiple specialists). We tracked data on each of our research questions for each of the individual providers the consumer reported accessing (e.g., how that consumer accessed each specialist, what referral pathway was used for each specialist and what treatments each specialist suggested). Whenever data were not explicitly reported in a consumer interview to answer these questions, we recorded it as missing data.

We compared HCP and consumer‐reported management practices to the guideline recommendations for chronic pain management in the Pain and Analgesia section of the Australian Therapeutic Guidelines [3]. These guidelines are generated by an expert group with input from guideline users and key stakeholder groups, they are renewed every 4 years, and are commonly integrated into Australian medical training programmes. The Australian Therapeutic Guidelines recommend taking a thorough assessment, providing patient education, generating management plans and maintaining a therapeutic relationship [3]. These guidelines recommend prioritising patient engagement with first‐line, nonpharmacological management strategies (i.e., increasing physical activity and social connection, addressing thoughts and emotions and improving nutrition and sleep), with second‐line management options including analgesics and invasive procedures [3]. The guidelines also include management options with questionable efficacy (e.g., massage, acupuncture, spinal manipulation) [3]. A more detailed presentation of these recommendations is included in File S4, which provides Supporting Information for our results. To compare the data, we generated an Excel spreadsheet including a tab for each section of the guideline recommendations, with columns generated for the specific recommendations in each section. We generated rows for each interviewed HCP and consumer with their reported data regarding the number of responses for each recommendation from each provider type entered in relevant columns.

After finishing HCP data collection and extraction, and halfway through consumer data collection and prior to starting consumer data extraction, we invited one of the interviewed consumers [MW] and HCPs [CR] to join the research team. This was a deviation from protocol, conducted to strengthen the interpretation of our results with the help of individuals with lived experience managing chronic pain in rural Australia. Author MW contributed to data extraction, so we removed her interview transcript from the analysis. Author CR was not involved in participant level data extraction and analysis, so his interview data were retained.

A researcher with previous qualitative content analysis experience and knowledge of chronic pain management [AM] extracted data from 30% of HCP transcripts with ARG. The rural chronic pain consumer [MW] extracted data from 30% of consumer transcripts with ARG. The pairs [AM+ARG and MW+ARG] extracted data from one transcript together, then independently extracted data from remaining transcripts for their 30% and met to compare and to update the reference document. Agreement on data extraction was reached when the pairs went through two interview transcripts without making additional changes to the reference document. Once agreement was reached, a single author [ARG] extracted data from the remaining transcripts utilising the reference document. Additionally, we descriptively analysed quantitative demographic data in Excel.

## 3 Results

We conducted interviews with 15 HCPs and 27 consumers between October 2022 and May 2023. This number of consumer interviews was a deviation from protocol where we proposed to interview 15 consumers to gain diverse perspectives. When we reached this threshold, 80% of interviewed consumers resided in South Australia. To increase the diversity of perspectives gained, we aimed to include interviews with two consumers from each of the other Australian states/territories. Demographic details of included participant interviews are presented in Table 1.

**TABLE 1:** Healthcare professional and consumer demographic data.

	HCP (*n* = 15)	Consumers (*n* = 27)
Sex (assigned at birth) [*n* (%)]		
Female	3 (20%)	18 (69%)
Male	12 (80%)	7 (27%)
Not listed	0	1 (4%)
Gender [*n* (%)]		
Female	3 (20%)	18 (69%)
Male	12 (80%)	7 (27%)
Not listed	0	1 (4%)
Age [mean, SD]	42.7 (8.4)	59.3 (12.6)
Australian state or territory [*n* (%)]		
South Australia	11 (73%)	11 (42%)
Victoria	1 (7%)	4 (15%)
Queensland	0	4 (15%)
New South Wales	0	3 (12%)
Tasmania	0	2 (8%)
Northern Territory	0	1 (4%)
Western Australia	3 (20%)	1 (4%)
Modified monash model classification [*n* (%)]		
MM2	0	4 (15%)
MM3	7 (47%)	5 (19%)
MM4	8 (53%)	14 (54%)
MM5	0	3 (12%)
Clinical discipline [*n* (%)]		
General practitioner	9 (60%)	Not applicable
Physiotherapist	4 (27%)	
Hospital doctor	2 (13%)	
Years worked clinically [*n* (%)]		
0–4 years	0	Not applicable
5–9 years	4 (27%)	
10–14 years	3 (20%)	
15–19 years	4 (27%)	
20+ years	4 (27%)	
Years (in total) worked in a rural, regional or remote Australian setting [*n* (%)]		
0–4 years	3 (20%)	Not applicable
5–9 years	6 (40%)	
10–14 years	2 (13%)	
15–19 years	1 (7%)	
20+ years	3 (20%)	
Reside in same rural, regional or remote location that they work in as a HCP (yes) [*n* (%)]	14 (93%)	Not applicable
Region of birth [*n* (%)]		
Oceania (Australia)	7 (47%)	Not applicable
Asia (Malaysia, Sri Lanka, Singapore)	4 (13%)	
Africa (Nigeria, Zimbabwe, Kenya)	3 (7%)	
Europe (UK)	1 (7%)	
Highest level of education [*n* (%)]		
Some high school or less	Not applicable	2 (8%)
Completed high school		8 (31%)
TAFE/certificate/Diploma		6 (23%)
University		7 (27%)
University higher degree (Masters, PhD)		3 (12%)
How managing financially [*n* (%)]		
Living comfortably	Not applicable	9 (35%)
Getting by		9 (35%)
Finding it difficult		8 (31%)
Current main activity [*n* (%)]		
Unemployed	Not applicable	1 (4%)
Working—full time		4 (15%)
Working—part time/casual		2 (8%)
Unable to work (disability/work cover/carer)		8 (31%)
Home duties		1 (4%)
Retired		10 (38%)

### 3.1 Pain Care Accessed by Consumers

All consumers reported accessing a GP. The majority reported accessing an allied health professional (89%). The four most frequently seen allied health professionals were physiotherapists (74%), exercise physiologists (41%), chiropractors (30%) and psychologists (30%). Two‐thirds (67%) of consumers consulted specialists, with the four most frequently seen specialists being pain specialists (30%), back specialists (19%), neurologists (15%) and neurosurgeons (15%). Seventy‐eight percent of consumers accessed a Complementary and Alternate Medicine provider, with the top three most frequently seen Complementary and Alternate Medicine provider types being massage therapists (56%), acupuncturists (26%) and medical cannabis companies (11%). One‐third (33%) of consumers reported accessing a multidisciplinary pain service. Table 2 presents the percentage of interviewed consumers who reported accessing each HCP category and the average rate of those consumers accessing each category.

**TABLE 2:** Consumer‐reported access to healthcare professional categories.

Healthcare professional category	Number and percentage of consumers who accessed each service (*n* = 27)	Average rate of number of providers of this type seen by consumers who accessed this provider type
General practitioners	27 (100%)	1.5
Allied health professionals^a^	24 (89%)	2.8
Complementary and alternate medicine providers^b^	21 (78%)	1.7
Specialists^c^	18 (67%)	2.7
Multidisciplinary pain services	9 (33%)	1.0
Emergency departments	3 (11%)	1.0

^a^ Allied health professionals included physiotherapists, exercise physiologists, chiropractors, psychologists, osteopaths, podiatrists and an occupational therapist.

^b^ Complementary and Alternate Medicine providers included massage therapists, acupuncturists, medical cannabis companies, Feldenkrais therapists, a Bowen therapist, and a myotherapist.

^c^ Specialists included back, shoulder, knee, and pain specialists, orthopaedic surgeons, neurologists, neurosurgeons, rheumatologists, gynaecologists, psychiatrists, urologists and endocrinologists.

### 3.2 Healthcare Professional and Service Accessibility

The services that consumers were most likely to access locally included a GP, an allied health professional, or a Complementary and Alternate Medicine provider. Consumers were most likely to travel to access specialists and multidisciplinary pain services. Consumers infrequently described accessing services via telehealth. The telehealth services that consumers accessed were often reported as occurring during COVID‐19 when they found their telehealth access improved:

I would say that while obviously, the whole lockdown situation was bad overall for the general population, for me, it actually opened up more opportunities. With the telehealth coming online, all of a sudden, I could actually access services that I couldn't access before. (Consumer, Female, Queensland)

**FIGURE 1:**
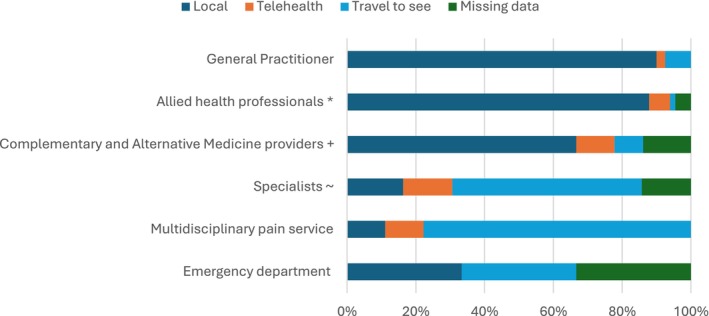
How rural consumers accessed different healthcare professional (HCP) types. * = Allied health professionals included physiotherapists, exercise physiologists, chiropractors, psychologists, osteopaths, podiatrists and an occupational therapist; + = Complementary and Alternative Medicine providers included massage therapists, acupuncturists, medical cannabis companies, Feldenkrais therapists, a Bowen therapist and a myotherapist; ~ = Specialists included back, shoulder, knee and pain specialists, orthopaedic surgeons, neurologists, neurosurgeons, rheumatologists, gynaecologists, psychiatrists, urologists and endocrinologists. 0% 20% 40% 60% 80% 100% General Practitioner Allied health professionals * Complementary and Alternative Medicine providers + Specialists ~ Multidisciplinary pain service Emergency department Local Telehealth Travel to see Missing data.

These findings are illustrated in more detail in Figure 1 and full details of the data are provided in File S3.

### 3.3 Access Pathways for Pain Care

**FIGURE 2:**
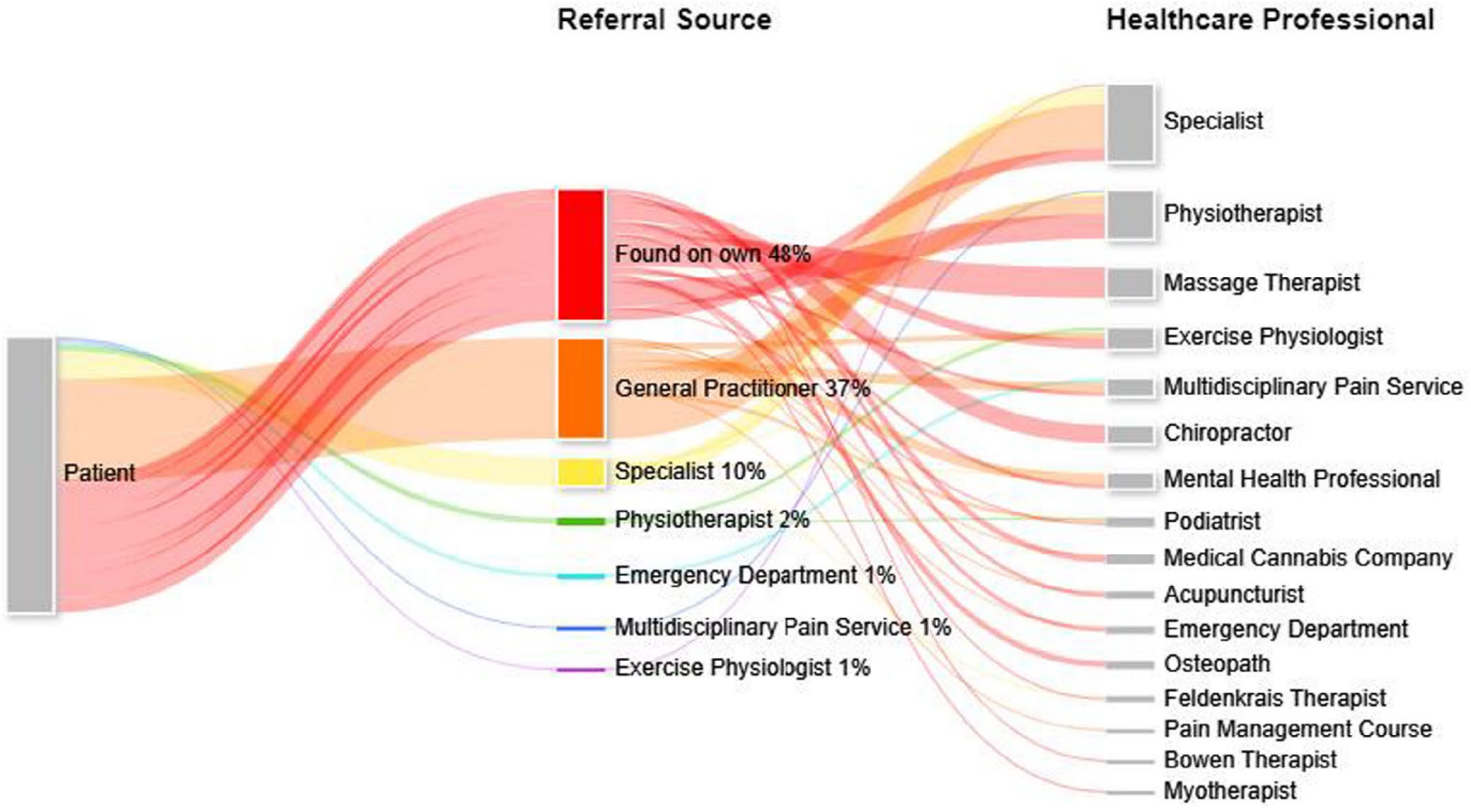
Consumer‐reported pathways utilised to access healthcare professionals for pain care.

Consumers reported finding almost half of the HCPs and services they accessed for pain care on their own and in all cases sought the care of chiropractors, osteopaths and massage therapists on their own. Consumers described that their GPs were most likely to suggest referrals to a specialist or an allied health professional, and specialists were most likely to suggest referrals to another specialist. Consumer‐reported access pathways for pain care are illustrated in Figure 2, and a detailed summary of the data is provided in File S3.

Table 3 presents the outbound referral pathways reported by HCPs. When asked, most GPs described not having telehealth referral pathways aside from two who referred to telehealth psychology services and two who described their only telehealth service use was when a specialist did telehealth consultations alongside in person support:

The telehealth services I have referred to are more or less an initial consultation prior to formally being reviewed by the pain specialists… that would be the only instance where I have used telehealth. (GP, South Australia)

**TABLE 3:** Healthcare professional outbound referral pathways.

Referrer	Refer to multidisciplinary pain service	Refer to allied health professional	Refer to specialist	Refer to community activities	Other
General practitioner (nine total)	9 (100%)	8 (89%)	8 (89%)	0	0
Hospital doctor (two total)	1 (50%)	1 (50%)	0	0	0
Physiotherapist (four total)	0	4 (100%)	1 (25%)	1 (25%)	1 (25%)

## 4 Healthcare Professional‐Reported Pain Management Practices Compared With Guideline Recommendations

We identified inconsistencies in adherence to first‐line management amongst HCPs involved in this study. HCPs regularly described applying recommended first‐line management strategies of addressing thoughts and emotions and promoting increased physical activity. However, they infrequently mentioned addressing other recommended first‐line management strategies, such as improving social connection, nutrition and sleep. In line with the guidelines, HCPs infrequently described reliance on second‐line management strategies of analgesics or invasive procedures. The most common second‐line management strategy described by HCPs with prescribing rights was the prescription of opioids (82%). Despite the high percentage of HCPs mentioning prescribing opioids for pain management, HCPs also reported avoiding prescribing opioids:

Avoiding very much opiates whenever we can. (GP, Western Australia)

If they are coming for something that they shouldn't need anything stronger than simple analgesia for, and that's the first time they're known to our clinic, they will not be getting opioids from me, because otherwise you find they come back. (GP, Western Australia)

Most HCPs, including all physiotherapists, reported implementing guideline recommendations to educate patients about chronic pain. Additionally, some HCPs were aware of the importance of developing a therapeutic relationship and collaborated with their patient to generate management plans. Several HCPs described facing challenges maintaining a therapeutic relationship and delivering guideline‐based care, as indicated by the below quote:

In 15‐minute consults, it's hard to get all that going really well [i.e., guideline‐based care] and to keep them coming back, too. Because if someone in the next room is going to give them Targin [an opioid medication], then why would you start learning how to breathe… [other providers' opioid prescribing] really is quite destructive, all that work you do on building a relationship and trust and engagement, and that's squashed by one consult that's taken them three minutes. (GP, South Australia)

We present further details of HCP‐reported routine pain management practices alongside guideline recommendations in tables in File S4.

## 5 Consumer‐Reported Pain Management Practices Compared With Guideline Recommendations

Despite guidelines recommending only ordering imaging in certain circumstances, 41% of consumers mentioned being referred for imaging, and 19% were referred for repeat scans. One participant described:

They just thought, ‘well let's just do scans’ because we just couldn't find where the pain was. We were hunting for the reason for the pain. And look, I'll be honest, I think we tallied up probably $6000 in scans that I paid for. (consumer, Female, Queensland)

Recommended first‐line management strategies of addressing thoughts and emotions and promoting increased physical activity were frequently reported by consumers. The provider types most frequently described as offering these two strategies were the provider types whose job description it is to do this work (i.e., psychologists and psychiatrists addressing thoughts and emotions; and physiotherapists and exercise physiologists increasing physical activity). Alternatively, no consumers described first‐line management strategies of increasing social connection or improving sleep, and only 7% of consumers described nutrition support as part of their received pain care.

Consumers described moderate use of second‐line management strategies of analgesics and invasive procedures. Several consumers detailed challenges gaining opioid prescriptions:

I usually have to beg, even though the pain specialist has said I can have them… I get a bit frustrated when I have to beg. (consumer, Female, Victoria)

Despite guidelines recommending against prescribing cannabinoids for chronic pain, 22% of consumers described trialling cannabinoids for pain management. Half of these cannabinoid prescriptions originated from the consumer requesting this treatment from their provider and the other half described a HCP recommended trialling this treatment.

While current guidelines list acupuncture, massage and passive mobilisation or spinal manipulation as having "uncertain benefit and potential harm [3], consumers frequently described receiving these treatments from allied health professionals and Complementary and Alternate Medicine providers. Consumers also infrequently described guideline‐recommended education and management plans as part of their received pain care.

Many consumers described positive experiences of therapeutic relationships with HCPs, including 44% and 46% of consumers who accessed GPs and allied health professionals, respectively, describing positive aspects of their relationships. Additionally, 63% of consumers described negative HCP therapeutic relationship experiences. Descriptions of negative experiences included perceiving their provider to be ‘useless’ or that their provider was unsure how to manage chronic pain (37%), they were dismissive of their pain (22%), and would not write prescriptions for opioids or only offered paracetamol (11%).

We present further details on consumer reports of received pain care alongside guideline recommendations in tables in File S4.

## 6 The Gap Between Care Provided for Chronic Pain and Care Recommended by Guidelines for Chronic Pain in Rural Australia

We identified a gap in rural consumers' local access to specialists and multidisciplinary pain services, including poor use of telehealth services that would decrease consumer's travel burden. Additionally, many consumers reported needing to identify the pain care services they wanted to be referred to themselves, rather than their providers initiating referrals to pain care services. HCPs provided insight into possible reasons for this evidence‐practice gap by reporting that desired services were not readily available, and specifically—they described not having access to specialists or psychologists. Some HCPs indicated visiting services stopped during the COVID‐19 pandemic and had not re‐commenced.

A deviation from recommended practice was found in both HCP and consumer reports of minimal use of first‐line management strategies of improving social connection, nutrition and sleep. Both participant groups also described challenges maintaining therapeutic alliances. Consumer reports of received care presented additional gaps including frequent imaging, use of cannabinoids for pain management, and not receiving education or management plans. Additionally, consumers frequently described receiving treatments with questionable efficacy, particularly from services they sought access to on their own.

In Table 4, we detail the gaps identified between what care the guidelines recommend for chronic pain and what care rural HCPs and consumers described for chronic pain.

**TABLE 4:** The gap between the Therapeutic Guidelines [3] recommendations and reports of care provided for chronic pain in rural Australia.

Guideline recommendation	Findings from reports of delivered and received care
Referrals can be used to address patient's needs	Minimal reports of accessing specialist and multidisciplinary pain services locally Frequent reports of travelling to access these services Minimal reports of telehealth to decrease the travel burden on rural Australians Frequent HCP reports of lack of local access to desired services Frequent reports of consumers identifying services they want to access rather than HCPs initiating referrals
First‐line pain management strategies include improving social connection, sleep and nutrition	Minimal reports of first‐line pain management strategies of social connection, improving nutrition and improving sleep
Assessments should include review of previous investigations to determine if further investigations are warranted, with recommendations for low back pain, neck pain and neuropathic pain indicating a lack of utility of imaging	Frequent consumer reports of referrals for imaging
Cannabinoids are not recommended for chronic pain management due to potential harms outweighing any unlikely benefit	Frequent consumer reports of trialling cannabis
Acupuncture, dry needling, massage, passive mobilisation and spinal manipulation are classified as having ‘uncertain benefit and potential harm’ [3]	Frequent consumer reports of receiving acupuncture, dry needling, massage, passive mobilisation and spinal manipulation
Patient education is a key component of chronic pain management	Infrequent consumer reports of receiving pain education
‘Every patient with chronic pain should have an individualised pain management plan’ [3]	Infrequent consumer reports of management plans
Maintaining a therapeutic relationship is a key role of the HCP in chronic pain management	Frequent HCP and consumer reports of challenges maintaining therapeutic relationships

Abbreviation: HCP, healthcare professional.

## 7 Discussion

We interviewed rural HCPs and consumers to gain an understanding of how chronic pain is managed in rural Australia, including investigation of gaps between what guidelines recommend for the management of chronic pain and current practice in rural Australia. All interviewed consumers received care from a GP for pain management, and more than two‐thirds of consumers accessed allied health professionals, specialists or Complementary and Alternate Medicine providers. Consumers were most likely to access a GP, an allied health professional, or a Complementary and Alternate Medicine provider locally, and to travel to access specialists and multidisciplinary pain services, with few utilising telehealth services. We also identified that almost half of the services consumers reported accessing were found on their own, with GP's most likely to initiate referrals to specialists or allied health professionals.

In comparing HCP and consumer reports to guideline recommendations, we identified mixed findings including differences between HCP and consumer reports. HCPs described delivering pain care as aligned with guidelines including the provision of education to patients and the generation of management plans; consumers did not describe these aspects in their received pain care. Consumers described receiving care that diverged from the guidelines including referrals for imaging, prescription of cannabinoids and frequent use of treatments with questionable efficacy; HCPs did not describe the provision of these aspects as part of their pain care.

### 7.1 Comparison to Existing Literature

Our findings align with previous Australia‐wide studies of pain management practices, which identified high rates of imaging and medication use, and underutilisation of recommended first‐line management strategies [10, 11]. Additionally, a previous report of rural Australian GP's chronic pain management found higher rates of medication prescription and lower rates of referrals in rural than in metropolitan settings [15]. This also aligns with our findings of frequent mentions of opioid prescription and consumers describing having found many of their accessed healthcare services on their own. One suggested explanation for the high reliance on medications for pain management in rural settings is the lack of access to pain care services [24].

In light of poor rural service access, the minimal reported use of telehealth services was surprising, particularly given the recent required rise in telehealth utilisation due to the COVID‐19 pandemic [25]. Australian government policy changes that were implemented in 2020 to enable Medicare billing for telehealth services were made permanent for allied health professionals in January 2022 [26] and non‐GP medical specialists in July 2022 [27]. Given interviews were conducted from October 2022, HCPs should have been aware of the permanency of telehealth billing, yet consumers in our study reported minimal referrals to telehealth services. In prepandemic data, consumer level factors of low education level and low socioeconomic status negatively influenced access to telehealth [28]. Being middle age, having broadband access and good digital literacy have been reported to positively influence access to telehealth [28]. Given that data and that the majority of our consumer sample held at least a high school degree, did not describe financial hardship, and were almost all within the middle‐age range, a greater use of telehealth may have been expected. Additionally, prepandemic financial and regulatory barriers blocking telehealth use seem to have been overcome by the recent policy changes. Other barriers such as connectivity and technology challenges, digital literacy and consumer concern for a lesser quality of therapeutic relationship over telehealth than in person, may offer some explanations [29].

One explanation for the gap between HCPs' reported pain care (aligned with guidelines) and consumers' reported care (divergent from guidelines) could be that consumers do not recognise guideline care as relevant to their pain. Consumers have been found to expect a ‘magic pill’, ‘cure’ or something to be done to them to relieve their pain when they seek healthcare for pain management [30]. Consumers also frequently report a perceived need for an accurate diagnosis of their pain [31] and request staying on pharmacological pain management because they do not think nonpharmacological strategies are beneficial [32]. This aligns with our findings that many of the services consumers sought on their own offer manual therapies, which also may align with the minimal reports of telehealth usage. Given these reports regarding consumer expectations for pain care, it seems possible that, when consumers in our sample were asked about the pain care they received, they may not have considered assessment taking, provision of education or use of management plans to be relevant. In contrast, they may have considered imaging and prescriptions to be part of pain care. This line of thought aligns with our finding that consumers described begging for opioids, requesting cannabinoids and not being happy when their provider would not prescribe an opioid or only offered paracetamol. Consumers having expectations contrary to guideline recommendations has been reported to be challenging for HCPs trying to deliver guideline‐based pain care [33, 34]. This may be particularly challenging for rural HCPs to navigate given HCP and consumer relationships often exist outside of consults as well [35, 36]. This finding lends support to recent calls for ‘whole of community’ education in order to shift consumer expectations and community norms around chronic pain and how best to manage it [37].

### 7.2 Strengths and Limitations

Our study benefited from gaining both HCP and consumer perspectives to enable a more complete presentation of how pain is managed in rural Australia. Within our consumer sample, we gained input from consumers who identified as having chronic pain rather than focusing on a single condition. This broadened the range of perspectives we gained regarding the management of chronic pain conditions in rural Australian settings. We were also able to gain consumer representation from all states, though we failed to reach our recruitment target of two consumers from Western Australia, Australian Capital Territory and Northern Territory due to recruitment difficulties.

There are some important limitations of this study. Any design that relies on self‐report data introduces the potential of recall and reporting bias. We explored how pain is managed using open‐ended questions, without specific probing for each item in the guidelines, which may explain low reporting of individual aspects of guideline care (see Supporting Information for full information). For example, it is possible that HCPs infrequently described some first‐line management strategies as part of their routine pain care because their patients do not regularly consider social connection, nutrition or sleep to be problems for them. Further, we recruited 15 HCPs who had worked in a rural, regional or remote setting for a minimum of 3 months through networks of the research team, most of whom had an interest in pain care and were from South Australia. This approach introduced recruitment bias and may have resulted in less representative data than a larger, Australia‐wide sample would have provided. This small‐scale study was considered acceptable because it laid the groundwork for later elements of the needs assessment, and we did not seek to recruit a nationally representative sample. Although there are no differences in applicable guideline care for chronic pain between states, there may be systemic or capability differences between states that were not captured here. Our HCP sample was primarily composed of GPs and physiotherapists, which presents a limitation in the diversity of HCP perspectives gained. Additionally, consumers in our sample were not patients or clients of the interviewed HCPs. We acknowledge that our consumer sample may have overrepresented those with polarising positive or negative experiences of pain care. It is also possible that the HCPs involved may have been those more familiar with guideline recommendations for pain, however, our findings indicate that this was unlikely the case based on identified inconsistencies between guideline recommendations and described care.

### 7.3 Implications and Future Directions

Our findings indicate that current care practices for chronic pain in rural settings inconsistently align with guideline‐based care. This inconsistency in care may be associated with higher patient pain ratings and healthcare expenditure [38]. Previous literature has indicated that short appointment times are likely to affect rural HCP delivery of guideline‐based pain care [39, 40, 41]. Short appointment times likely impact providers' ability to thoroughly assess and discuss the biopsychosocial aspects of a patient's pain, to effectively deliver pain education and to collaboratively generate management plans. Future research and policy action to improve rural pain care could investigate strategies for increasing appointment times or alternative strategies to combat this challenge. One such alternative strategy could be to trial group appointments comprised of consumers with common health challenges. Additionally, future work could promote a shift in community norms and consumer expectations of pain care, with one suggested approach being to investigate the effect of whole of community pain education.

Our findings also suggest that increasing access to pain services has the potential to improve the implementation of guideline‐based pain care, and pain outcomes in rural settings. Specifically, services that support pain education and first‐line management strategies aimed at improving sleep, nutrition and social connection need to be more readily available. HCPs who are not specialised in these areas can discuss these aspects of care with a patient, but without services to refer them to for support, improving these aspects of a patient's life remains difficult. While it is unlikely to be possible to increase local service access in all rural areas, one solution could be increased access to telehealth services and support to increase telehealth uptake. In line with increasing uptake, strategies should be trialled to improve both HCP and consumer awareness of available services. One way to enhance HCP awareness of available services could be generation of locally relevant standardised care pathways to inform HCP referrals to guideline‐recommended services. Additionally, areas affected by ongoing health workforce challenges may benefit from pain and lifestyle education programmes delivered by non‐HCPs.

## 8 Conclusions

There are critical gaps between present pain care in rural Australia and the care recommended by guidelines. These gaps include poor use of telehealth services that may be able to fill service provision challenges and consumers relying on their own investigations to identify services to support their pain management. Additionally, we identified minimal usage of recommended first‐line management strategies, and common receipt of nonguideline care, including imaging, manual therapies and prescription medications. Future efforts to improve rural pain care may benefit from improving access to pain care services, developing approaches for shifting consumer expectations for care and overcoming short appointment times.

## Ethics Statement

This project was approved by the University of South Australia's Health Research Ethics Committee (protocol #204795).

## Author Contributions

**Ashley R. Grant:** conceptualization, methodology, investigation, resources, formal analysis, visualization, writing – original draft, writing – review and editing, project administration. **Monique White:** data curation, formal analysis, writing – review and editing. **Amelia Mardon:** formal analysis, writing – review and editing. **Peter D. Hibbert, Emma L. Karran, and Gill Westhorp:** conceptualisation, writing – review and editing, supervision. **Christopher Roeger:** formal analysis, writing – review and editing. **G. Lorimer Moseley:** conceptualization, writing – review and editing, supervision, funding acquisition.

## Conflicts of Interest

G.L.M. has received support from Reality Health, ConnectHealth UK, AIA Australia, Institutes of Health, California, Kaiser Permanente, Workers' Compensation Boards in Australia, Europe and North America, the Melbourne Football Club. G.L.M. receives royalties for several books on pain and speakers' fees for talks on pain, pain education, physiotherapy and rehabilitation. A.R.G., G.W., A.M., M.W., P.H., E.L.K., C.R. and G.S. declare no competing interests.

## Supporting information


**Data S1.** Supporting Information.

## Data Availability

Research data are not shared.
