# Supplementary material for: How Is Chronic Pain Managed in Rural Australia? A Qualitative Study Exploring Rural Healthcare Professional and Consumer Experiences
Source: Aust J Rural Health. 2025 Feb 10;33(1):e70000. doi: 10.1111/ajr.70000 (PMC11809133; doi:10.1111/ajr.70000)
Supplement: Supplementary file 1 — Data S1. Supporting Information. [file AJR-33-0-s001.docx]

**Supplementary File 1**

Modified Monash Model adapted from the Modified Monash Model Fact Sheet ^1^

| **Modified Monash Model Category** | **Definition** |
| --- | --- |
| *MM1* | Metropolitan – major cities |
| *MM2* | Regional centres – regional areas in or within a 20 km drive of a town with a population over 50,000 |
| *MM3* | Large rural towns – regional areas that are not classified as MM2 and are in or within a 15 km drive of a town with a population of 15,000-50,000 |
| *MM4* | Medium rural towns – regional areas that are not classified as MM2 or MM3, and are in or within a 10 km drive of a town with a population of 5,000-15,000 |
| *MM5* | Small rural towns – all remaining regional areas |
| *MM6* | Remote communities – remote areas that are mainland or islands less than 5 km offshore |
| *MM7* | Very remote communities – very remote areas |

1. *Modified Monash Model - fact sheet*. 2019. <https://www.health.gov.au/resources/publications/modified-monash-model-fact-sheet?language=en>

**Supplementary File 2**

**Clinician Interview Topic Guide**

***Section 1 – Describe yourself and your rural work environment***

- Can you describe your rural clinical practice setting?
  - Additional prompting questions:
    - Do you work in a single speciality or multidisciplinary clinic?
    - How many patients do you see on an average day?
    - How long are your consults?
- Roughly, what percentage of your patients present with chronic pain?

***Section 2 – Describe how you manage patients with persistent pain* in your rural workplace location**

- What strategies do you use most often when working with patients with chronic pain?
  - Additional prompting questions:
    - What do you think has led to you to manage chronic pain with these strategies?
    - Have your chronic pain management strategies changed over the years? How so?
    - If you also work or have worked in a metro location or another rural clinical practice location, did your chronic pain management strategies differ in that setting? How so?
- Can you describe any referral pathways you use for patients with chronic pain?
  - Additional prompting questions:
    - How do patients come to work with your services (inbound referral pathways)?
    - What services do you refer patients to (outcome referral pathways)?
    - How have you come to refer to these specific services?
    - Why have you chosen to use those services?
    - Do you refer to any telehealth services? Online services? Community organisations?
    - Have your referral pathways changed since the COVID-19 pandemic?

**Consumer Interview Topic Guide**

***Section 1 – Describe the healthcare you have received for pain management***

- What local healthcare professionals have you seen for assistance with managing for your chronic pain?
  - Follow up questions for each healthcare professional mentioned:
    - How did you come to access this service? Did someone refer you to see them or did you seek them out yourself?
    - What treatments did this service offer you?
- Have you seen any healthcare professionals via telehealth due to how far away they are from you? If so, which providers have you worked with via telehealth?
  - Follow up questions for each healthcare professional mentioned:
    - How did you come to access this service? Did someone refer you to see them or did you seek them out yourself?
    - What treatments did this service offer you?
- Have you travelled to other areas to see healthcare professionals for assistance with managing your chronic pain? If so, which providers have you worked with via traveling to see them?
  - Follow up questions for each healthcare professional mentioned:
    - How did you come to access this service? Did someone refer you to see them or did you seek them out yourself?
    - What treatments did this service offer you?
- Additional follow up questions based on gaps in their responses:
  - Have you worked with any of the following services for support managing your pain:
    - A GP?
    - Physiotherapist?
    - Exercise physiologist?
    - Chiropractor?
    - Osteopath?
    - Massage therapist?
    - Acupuncture?
    - Mental health professional?
    - Any specialists?
  - If yes, additional prompting questions with the following:
    - How did you come to access this service? Did someone refer you to see them or did you seek them out yourself?
    - What treatments did this service offer you?

**Supplementary File 3**

Data for Figure 1. How rural consumers are accessing different healthcare professional (HCP) types

|  | **Local** | **Telehealth** | **Travel to see** | **Missing data** |
| --- | --- | --- | --- | --- |
| *GP* (40) | 36 (90%) | 1 (3%) | 3 (8%) | 0 |
| *Allied health professionals†* (66) | 58 (88%) | 4 (6%) | 1 (2%) | 3 (5%) |
| *Specialists¶* (49) | 8 (16%) | 7 (14%) | 27 (55%) | 7 (14%) |
| *‘Other’ providers§*  (36) | 24 (67%) | 4 (11%) | 3 (8%) | 5 (14%) |
| *Multidisciplinary pain service* (9) | 1 (11%) | 1 (11%) | 7 (78%) | 0 |
| *Emergency Department* (3) | 0 | 0 | 1 (33%) | 2 (67%) |

† *= Allied health professionals included physiotherapists, exercise physiologists, chiropractors, mental health professionals, osteopaths, podiatrists, and an occupational therapist; ¶= Specialists included back, shoulder, knee, and pain specialists, orthopaedic surgeons, neurologists, neurosurgeons, rheumatologists, gynaecologists, psychiatrists, urologists, and endocrinologists; § = ‘Other’ providers included massage therapists, acupuncturists, medical cannabis companies, Feldenkrais therapists, a Bowen therapist, and a myotherapist*

Data for Figure 2. Consumer reported referral pathways

|  | **Patient found** | **GP referral** | **Specialist referral** | **Allied health referral** | **Other provider referral** | **Missing data** |
| --- | --- | --- | --- | --- | --- | --- |
| *Allied health professionals†*  (66) | 32 (48%) | 19 (29%) | 4 (6%) | 3 (5%) | 3 (5%) | 5 (8%) |
| *Specialists¶* (49) | 6 (12%) | 22 (45%) | 9 (18%) | 1 (2%) | 0 | 11 (22%) |
| *‘Other’ providers§* (36) | 22 (61%) | 3 (8%) | 1 (3%) | 0 | 0 | 10 (28%) |
| *Multidisciplinary pain service* (9) | 2 (22%) | 5 (56%) | 0 | 0 | 2 (22%) | 0 |
| *Emergency Department* (3) | 2 (67%) | 1 (33%) | 0 | 0 | 0 | 0 |

† *= Allied health professionals included physiotherapists, exercise physiologists, chiropractors, mental health professionals, osteopaths, podiatrists, and an occupational therapist; ¶= Specialists included back, shoulder, knee, and pain specialists, orthopaedic surgeons, neurologists, neurosurgeons, rheumatologists, gynaecologists, psychiatrists, urologists, and endocrinologists; § = ‘Other’ providers included massage therapists, acupuncturists, medical cannabis companies, Feldenkrais therapists, a Bowen therapist, and a myotherapist*

**Supplementary File 4**

Healthcare professional (HCP)-reported management practices compared with guideline recommendations

| **Guideline recommendations (from Therapeutic Guidelines^37^)** | **Reported care practices** | | |
| --- | --- | --- | --- |
|  | **General Practitioners**  **(n=9)** | **Allied Health Professionals**  **(n=4)** | **Hospital**  **Doctors**  **(n=2)** |
| Assessment | | | |
| Take a history of pain | 33% | 50% | 50% |
| Assess sociopsychobiomedical context | 11% | 25% |  |
| Assess patient’s experience and meaning of pain | 11% | 25% |  |
| Take a physical exam |  | 25% |  |
| Review previous investigations, decide if further testing is indicated | 11% |  | 50% |
| First-line management strategies | | | |
| Increase social connection |  | 25% |  |
| Address thoughts and emotions | 56% | 50% |  |
| Increase physical activity | 44% | 75% |  |
| Improve nutrition | 11% | 25% |  |
| Improve sleep | 11% |  |  |
| Second-line management strategies – analgesics | | | |
| Short term paracetamol | 22% |  |  |
| If paracetamol ineffective, short term NSAID | 33% |  |  |
| If indicated, adjuvants |  |  |  |
| If appropriate, short term (<12 weeks) opioid may be considered.  If longer, consult a specialist. | 89% |  | 50% |
| Do not recommend cannabinoids |  |  |  |
| Only pain specialists should recommend methadone & hydromorphone |  |  |  |
| Second-line management strategies – invasive procedures | | | |
| Percutaneous radiofrequency neurotomy |  |  |  |
| Neuromodulation |  |  |  |
| Epidural block |  |  |  |
| Management strategies with questionable efficacy | | | |
| Acupuncture |  |  |  |
| Dry needling |  |  |  |
| Graded motor imagery |  |  |  |
| Hot and cold superficial therapy |  |  |  |
| Massage |  | 25% |  |
| Passive mobilisation / spinal manipulation |  |  |  |
| TENS |  |  |  |
| Patient education | | | |
| Educate about chronic pain | 56% | 100% |  |
| Management Plans | | | |
| Generate management plan | 22% | 75% |  |
| Include goal setting | 22% | 25% |  |
| HCPs’ roles in pain management | | | |
| Maintain a therapeutic relationship | 11% | 25% |  |


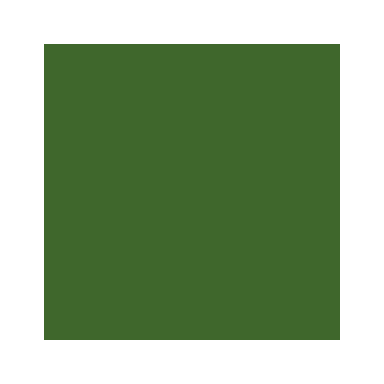
=≥67%,
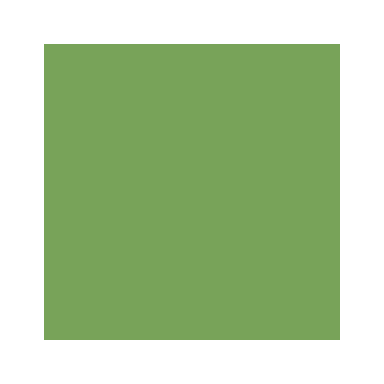
=34-66%, and
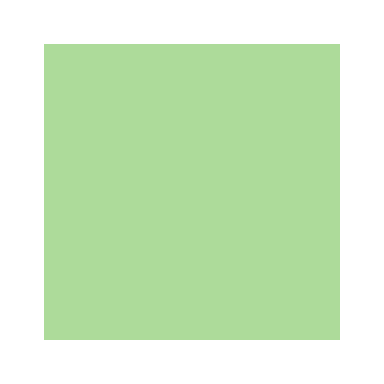
=1-33% of HCPs reported using this guideline-described strategy

Healthcare professional (HCP)-reported management practices not described in the guideline recommendations

| **Guideline category - Treatment mentioned that is not in the guidelines** | **Reported care practices** | | |
| --- | --- | --- | --- |
|  | **General Practitioners**  **(n=9)** | **Allied Health Professionals**  **(n=4)** | **Hospital**  **Doctors**  **(n=2)** |
| Second-line management strategies – invasive procedures | | | |
| Steroid injections | 11% |  |  |
| Nerve blocks | 11% |  | 50% |

Consumer-report of healthcare professional (HCP) management practices compared with guideline recommendations

| **Guideline recommendations (from Therapeutic Guidelines^37^)** | **Reported care received from each provider type** | | | | |
| --- | --- | --- | --- | --- | --- |
|  | **General Practitioner (n=27)** | **Allied Health Professional (n=24)** | **‘Other’ Providers**  **(n=21)** | **Specialists (n=18)** | **Multi-D Service (n=9)** |
| Assessment | | | | | |
| Take a history of pain |  |  |  |  |  |
| Assess sociopsychobiomedical context |  |  |  |  |  |
| Assess patient’s experience and meaning of pain |  |  |  |  |  |
| Take a physical exam |  |  |  |  |  |
| Review previous investigations, decide if further testing is indicated | 26% |  |  | 33% | 11% |
| First-line management strategies | | | | | |
| Increase social connection |  |  |  |  |  |
| Address thoughts and emotions | 7% | 42% | 5% | 22% | 11% |
| Increase physical activity | 15% | 88% | 14% | 17% | 11% |
| Improve nutrition | 4% |  |  | 6% |  |
| Improve sleep |  |  |  |  |  |
| Second-line management strategies – analgesics | | | | | |
| Short term paracetamol | 15% |  |  | 6% | 11% |
| If paracetamol ineffective, short term NSAID | 7% |  |  | 11% | 11% |
| If indicated, adjuvants | 11% |  |  | 11% |  |
| If appropriate, short term (<12 weeks) opioid may be considered.  If longer, consult a specialist. | 52% |  |  | 33% |  |
| Do not recommend cannabinoids | 15% |  | 14% |  | 11% |
| Only pain specialists should recommend methadone & hydromorphone |  |  |  | 11% |  |
| Second-line management strategies – invasive procedures | | | | | |
| Percutaneous radiofrequency neurotomy |  |  |  |  |  |
| Neuromodulation |  |  |  | 22% | 11% |
| Epidural block |  |  |  |  |  |
| Management strategies with questionable efficacy | | | | | |
| Acupuncture |  | 8% | 38% |  |  |
| Dry needling | 4% | 21% | 5% |  |  |
| Graded motor imagery |  |  |  |  |  |
| Hot and cold superficial therapy |  | 4% |  |  |  |
| Massage |  | 38% | 71% |  | 11% |
| Passive mobilisation / spinal manipulation |  | 42% |  |  |  |
| TENS |  | 4% |  |  | 11% |
| Patient education | | | | | |
| Educate about chronic pain |  | 8% | 5% |  |  |
| Management Plans | | | | | |
| Generate management plan | 7% |  |  |  |  |
| Include goal setting |  |  |  |  |  |
| HCPs’ roles in pain management | | | | | |
| Maintain a therapeutic relationship | 44% | 46% | 14% | 33% | 22% |


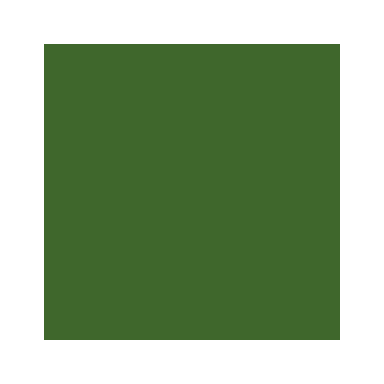
=≥67%,
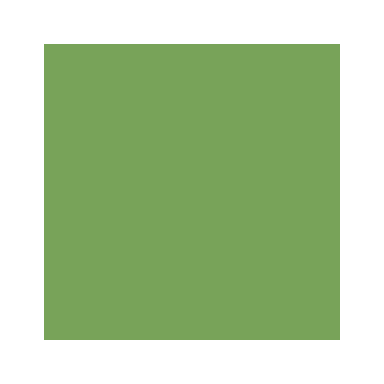
=34-66%, and
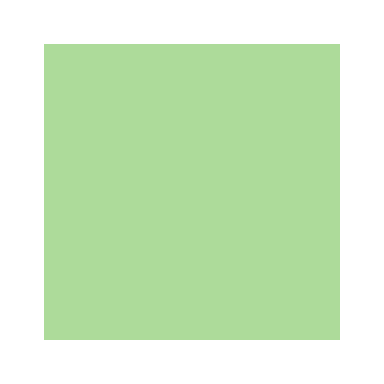
=1-33% of consumers who saw this provider type reported using this guideline-described strategy;
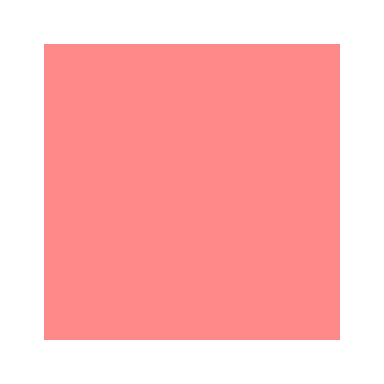
=a consumer reported management contradictory to the guideline recommendations

Consumer-report of healthcare professional (HCP) management practices not described in, or contrary to, the guideline recommendations

| **Guideline category - Treatment mentioned that is not in the guidelines** | **Reported care received from each provider type** | | | | |
| --- | --- | --- | --- | --- | --- |
|  | **General Practitioner (n=27)** | **Allied Health Professional (n=24)** | **‘Other’ Providers**  **(n=21)** | **Specialists (n=18)** | **Multi-D Service (n=9)** |
| Second-line management strategies – analgesics | | | | | |
| Birth control |  |  |  | 11% |  |
| Maxalt | 4% |  |  |  |  |
| Valium | 4% |  |  |  |  |
| Aminopyradine | 4% |  |  |  |  |
| Antispcyhotics |  |  |  | 6% |  |
| Biologics |  |  |  | 6% |  |
| Herbals and vitamins | 4% | 4% |  | 6% |  |
| Second-line management strategies – invasive procedures | | | | | |
| Steroid injections | 11% |  |  | 28% |  |
| Nerve blocks |  |  |  | 11% |  |
| Ketamine infusions |  |  |  | 22% |  |
| Surgery |  |  |  | 22% | 11% |
| Prolotherapy | 7% |  |  |  |  |
| Saline injections | 4% |  |  |  |  |
| Nerve ablation |  |  |  | 6% |  |
| Botox |  |  |  | 6% |  |
| A morphine pump |  |  |  |  | 11% |
| Management strategies with questionable efficacy | | | | | |
| Transcutaneous magnetic stimulation (TMS) |  |  |  | 6% |  |
| Orthotics |  | 13% |  |  |  |
| Cupping |  |  | 14% |  |  |
| Ultrasound |  | 4% |  |  |  |
| Inflatable neck extender |  |  |  | 6% |  |
| Left right judgement tasks |  | 4% |  |  |  |
| Shockwave therapy |  | 4% |  |  |  |
| A decompression machine |  | 4% |  |  |  |
| HCPs’ roles in pain management | | | | | |
| Negative experiences of therapeutic relationships | 56% | 13% |  | 22% | 11% |
